# Nutrition as an important mediator of the impact of background variables on outcome in middle childhood

**DOI:** 10.3389/fnhum.2013.00713

**Published:** 2013-10-25

**Authors:** Patricia Kitsao-Wekulo, Penny Holding, H. Gerry Taylor, Amina Abubakar, Jane Kvalsvig, Kevin Connolly

**Affiliations:** ^1^KEMRI/Wellcome Trust Research ProgrammeKilifi, Kenya; ^2^Department of Publications and Ethics, International Centre for Behavioural StudiesNairobi, Kenya; ^3^Discipline of Psychology, School of Applied Human Sciences, University of KwaZulu-NatalDurban, South Africa; ^4^Department of Research and Training, International Centre for Behavioural StudiesMombasa, Kenya; ^5^Case Western Reserve UniversityCleveland, OH, USA; ^6^Department of Pediatrics, Case Western Reserve University, Rainbow Babies and Children's Hospital, University Hospitals Case Medical CenterCleveland, OH, USA; ^7^Department of Child and Adolescent Studies, Utrecht UniversityUtrecht, Netherlands; ^8^Department of Cross-Cultural Psychology, Tilburg UniversityTilburg, Netherlands; ^9^Discipline of Public Health Medicine, School of Nursing and Public Health, University of KwaZulu-NatalDurban, South Africa; ^10^Department of Psychology, University of SheffieldSheffield, UK

**Keywords:** nutritional status, school-age children, structural equation modeling, direct and indirect effects, co-occurring risk factors, cognitive outcomes

## Abstract

Adequate nutrition is fundamental to the development of a child's full potential. However, the extent to which malnutrition affects developmental and cognitive outcomes in the midst of co-occurring risk factors remains largely understudied. We sought to establish if the effects of nutritional status varied according to diverse background characteristics as well as to compare the relative strength of the effects of poor nutritional status on language skills, motor abilities, and cognitive functioning at school age. This cross-sectional study was conducted among school-age boys and girls resident in Kilifi District in Kenya. We hypothesized that the effects of area of residence, school attendance, household wealth, age and gender on child outcomes are experienced directly and indirectly through child nutritional status. The use of structural equation modeling (SEM) allowed the disaggregation of the total effect of the explanatory variables into direct effects (effects that go directly from one variable to another) and indirect effects. Each of the models tested for the four child outcomes had a good fit. However, the effects on verbal memory apart from being weaker than for the other outcomes, were not mediated through nutritional status. School attendance was the most influential predictor of nutritional status and child outcomes. The estimated models demonstrated the continued importance of child nutritional status at school-age.

## Introduction

While the literature provides evidence that the negative effects of early malnutrition persist to school-age (Pollitt et al., 1996), there are several significant knowledge gaps. First, despite evidence that the impact of nutrition varies across different neurocognitive domains, there have been few studies investigating this area, especially in middle childhood. And yet at school age, children are exposed to more differential experiences and acquire more sophisticated abilities across various cognitive domains (Fischer and Bullock, 1984). Second, there is a complex inter-related relationship between poverty, nutritional status and neurocognitive outcomes. Not only do the constraints of low income in deprived settings create practical barriers to good nutrition; additional socio-environmental factors reinforce the effects of this deprivation (Engle and Black, 2008). Poor nutritional status at this age may have long-term negative consequences and restrict development of a child's full potential. This is therefore a critical period for investigating the link between malnutrition and developmental outcomes, especially within a multiple risk context.

In many developing countries, particularly in sub-Saharan Africa, linear growth retardation, or stunting, a manifestation of chronic protein-energy malnutrition (PEM), is highly prevalent, with rates as high as 38% (de Onis et al., 2012). Various individual and environmental variables have been associated with an elevated risk of experiencing poor nutritional status. Important differences have been highlighted in the prevalence of stunting among boys and girls (Badenhorst et al., 1993; Lwambo et al., 2000; Semproli and Gualdi-Russo, 2007; Acham et al., 2008; Omigbodun et al., 2010; Goon et al., 2011; Senbanjo et al., 2011) although there are substantial variations in regional trends. Moreover, patterns observed among school-age populations are similar to those reported at younger ages (Wamani et al., 2007). With regard to age, several studies have reported a dramatic increase in stunting among older children (Stoltzfus et al., 1997; Lwambo et al., 2000; Goon et al., 2011; Senbanjo et al., 2011) demonstrating that linear growth continues to falter throughout the school-age years (The Partnership for Child Development, 1998). Mendez and Adair (1999) found that children who started school at earlier ages (5 or 6 years) were substantially taller than children who started school later (7 or 8 years) so it may be that better-off children enrol in school at earlier ages. And although children in low income settings may all suffer the effects of deprivation, those from the least wealthy households in low income settings are more likely to be malnourished (Sigman et al., 1989; Brooks-Gunn and Duncan, 1997; Bradley and Corwyn, 2002; Abubakar et al., 2008; Ndukwu et al., 2013). Rural residence (Hautvast et al., 2000; Nabag, 2011) and a reduced likelihood of attending school (Ivanovic et al., 2012) have also been related to poor nutritional status. Over childhood, these risk factors have been known to alter the profile of undernutrition (protecting against or accentuating the risk of undernutrition) in a population (Pollitt et al., 1996), as well as being recognized as adversely affecting cognitive functioning independently of nutritional status.

Undernutrition has been shown to negatively impact on various developmental and cognitive domains including motor development (Pollitt et al., 1994; Chang-Lopez, 2007; Olney et al., 2007), language functioning (Wachs, 1995; Duc, 2009), IQ (Mendez and Adair, 1999) as well as memory and executive functions (Kar et al., 2008). This latter study observed that malnourished children showed poor performance on tests of higher cognitive functions but not on motor performance. Moreover, the impact of malnutrition on specific skills seems to vary according to diverse child-related and environmental variables. For instance, among the various gender-patterned deficits documented through an Indian study (Bhandari and Ghosh, 1980), malnutrition affected a wider range of aspects of immediate memory of boys than that of girls.

The effects and outcomes of nutritional status are correlated with environmental factors, the most salient of which is socioeconomic status (Bradley and Corwyn, 2002). Low SES leads to poor dietary intake which in turn impacts on brain and mental development eventually causing developmental deficits. School attendance has also been associated with better cognitive scores among both stunted and non-stunted children (Mendez and Adair, 1999). And as we have reiterated earlier on, rural children have a substantially higher risk of poor nutrition (Fox and Heaton, 2012) as well as poor cognitive outcomes.

In recent times, there have been efforts to investigate the complex relationship between background variables, nutritional status and developmental outcomes (Wachs, 1995). And in Kenya, a recent study investigated the direct and indirect effects of economic poverty on child outcomes (Abubakar et al., 2008). The results suggested that in infancy, impaired psychomotor development is associated directly with undernutrition, while the effect of poverty is mediated entirely through nutritional status (Abubakar et al., 2008). These results are similar to what had been earlier reported from Indonesia where nutritional influences mediated the relationship between poverty-related variables (e.g., SES) and child outcomes (Pollitt et al., 1994). As far as our literature search has revealed, the majority of studies exploring the relationship between undernutrition, co-occurring risk factors and other aspects of impaired child outcome has largely concentrated on children under the age of 5 years (Kariger et al., 2005; Abubakar et al., 2008; Olney et al., 2009, 2007; McDonald et al., 2013). We would like to build up on earlier work and extend the lines of research by focussing on school-age children.

Given the co-occurrence of malnutrition and multiple risk factors within this setting, are the adverse effects of these variables on neurocognitive outcomes related to their impact on nutritional status? Based on a model modified from Wachs (1995), we hypothesized that, (a) sociodemographic and biological factors make a unique contribution to nutritional status, and, (b) nutritional status is a strong predictor of various outcomes in school-age children. Because cognitive skills are more differentiated at this stage, we were able to explore the relationship between chronic malnutrition and developmental outcome across several outcomes. To delineate these effects and to investigate these relationships simultaneously required advanced statistical modeling. The main aim of this study was therefore to establish if diverse background characteristics created variations in nutritional status. We also sought to compare the relative strength of the effects of poor nutritional status on language skills, motor abilities, and cognitive functioning at school age. This information will enable the identification of points of intervention for those most at risk.

## Materials and methods

The study was cross-sectional in nature.

### Study setting

The study was conducted in Kilifi District, Kenya, among a predominantly rural community. The majority (66.8%) of the population lives below the poverty line and is therefore unable to access basic needs due to geographical, economic, and sociocultural barriers (Kahuthu et al., 2005). The district is a food deficit region relying on trade with other districts to meet the food gap—however, income-generating opportunities are few and unsustainable (FAO Kenya, 2007). Malnutrition remains rampant due to variability in crop production; and high illiteracy levels increase the population's vulnerability to food insecurity [Kenya National Bureau of Statistics (KNBS) and ICF Macro, 2010].

### Study sample

Children between the ages of 8 and 11 years were recruited from the catchment areas of five local primary schools distributed across neighborhoods ranging from sparsely populated rural areas to more densely populated semi-urban areas. The total sample of 308 children comprised both schooling and non-schooling children. Their first language was Mijikenda, the local vernacular or Kiswahili, the lingua franca and national language.

The Ten Questions Questionnaire (Mung'ala-Odera et al., 2004) was administered to parents to determine the presence of any impairments or serious health problems in children. When the parent was not able to determine if the child had any impairments (visual, auditory, or motor) or in cases where only milder concerns were reported, testing was attempted. Children who were physically unable to perform the tasks were excluded.

### Ethical considerations

The Kenya Medical Research Institute/National Ethics Review Committee (KEMRI/NERC) provided ethical clearance for the study. Permission to visit schools was obtained from the District Education Office. We explained the purpose of the study to the head teachers of selected schools and then sought their permission to recruit children. We also held meetings with community leaders, elders, and parents (and guardians) of selected pupils to explain the purpose of the study. After each meeting, a screening questionnaire was administered to establish if selected children met the study's eligibility criteria. We presented information on the study to parents in the language with which they were most familiar. We then obtained written informed consent for their children's participation. All the selected children assented to their participation in the study.

### Measurement of variables

**Figure 1:**
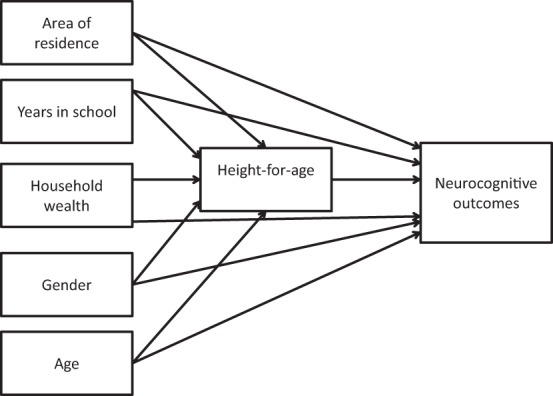
**Hypothesized model for testing the mediating influence of nutritional status on child neurocognitive outcomes**.

Building on the extant research literature, our analysis included age, gender, area of residence, school attendance and household wealth as underlying biological and environmental influences, nutritional status as a mediating variable and language skills, motor abilities and two factor scores of cognitive function as child outcomes. In order to test the various hypothesized relationships, we developed the model presented in Figure 1.

In the full model which included all the explanatory variables, the use of structural equation modeling (SEM) allowed the disaggregation of the total effect of the explanatory variables into direct effects (effects that go directly from one variable to another) and indirect effects (effects between two variables that are mediated by at least one intervening variable) (Bollen, 1989). We hypothesized that the effects of area of residence, school attendance, household wealth, age, and gender on child outcomes are experienced directly. Additionally, we hypothesized that the influence of these variables has an indirect effect on child outcomes through their influence on nutritional status. The model also took into account possible correlations among the five background variables. We fitted separate models for language skills, motor abilities, verbal memory, and executive function to see if there were differences among the four child outcomes.

#### Background characteristics

Information on child gender, age, school attendance (number of years that child has attended school), and household wealth was collected using a standard questionnaire. Birth records were used, where available, to confirm the child's date of birth. In the cases where records were not available, the procedure outlined by Kitsao-Wekulo et al. (2012) was followed. For the purpose of this study, an age variable in 6-month increments was created. An index of household wealth that divided the sample into three approximately equal groups—least wealthy (Level 1), moderately wealthy (Level 2), and the most wealthy (Level 3)—was derived from six socioeconomic indicators: maternal and paternal education, maternal, and paternal occupation, type of windows in the child's dwelling and ownership of small livestock. Area of residence was characterized as rural or peri-urban according to the most common settlement within the school catchment area.

#### Mediating factor

Children's heights were measured to the nearest centimetre using a stadiometer and height-for-age indices were calculated using EpiInfo (Centers for Disease Control, Atlanta, GA). Growth retardation was defined as height that was more than 2 standard deviations below levels predicted for age according to the World Health Organization reference curves for school-aged children (World Health Organization, 2007).

#### Child outcomes

A battery of neuropsychological tests was used to assess children's language skills, motor abilities, and cognitive functioning.

***Language skills.*** The Kilifi Naming Test (KNT), a test of confrontation naming, was used to assess expressive vocabulary (Kitsao-Wekulo et al., in preparation). In the KNT, the child was asked to spontaneously give one-word responses when presented with a black and while line drawing of a familiar object. Correct responses were coded “1.” A stimulus cue was provided when no response was given, the child stated that they did not know the name of the item or the item was perceived incorrectly. If the child did not provide a correct response after the stimulus cue, the word that was provided was recorded verbatim. The test was discontinued after six incorrectly named consecutive items. The final score was calculated by summing the number of spontaneously correct items and the number of correct items following a stimulus cue. These scores were standardized enabling the direct comparison of children's performance across tests.

***Motor abilities.*** Children's motor abilities were assessed using five tests of gross motor abilities covering two areas of motor performance—static and dynamic balance—and three timed tests of fine motor coordination and manual dexterity (Kitsao-Wekulo et al., under review). Age-corrected scores were obtained by computing differences between observed and predicted scores in units of standard error of the estimate (i.e., in z-score units). Maximum likelihood factor analysis with oblique rotation was then applied to the z-scores to reduce the multiple motor scores to ability composites (Ackerman and Cianciolo, 2000). Factor analysis yielded support for a two-factor solution; four tests loaded on the Motor-Co-ordination factor while the remaining four tests loaded on the Static and Dynamic Balance factor. Factor scores were defined as the mean of the z-scores for the tests loading on each factor. An Overall Motor Index was defined as the mean of the two factor scores.

***Cognitive functioning.*** We administered eight tests of cognitive functioning. These included:

- a non-verbal Tower Test of executive function to measure problem-solving and planning ability;
- the Self-Ordered Pointing Test (SOPT) to assess verbal/visual selective reminding in terms of the capacity to initiate a sequence of responses, retain the responses and monitor the consequences of behavior;
- Verbal List Learning (VLL) in which five serial verbal presentations of a 15-item word list were used to test learning and working memory;
- Dots, a non-verbal test of memory where the child was required to point at a special dot on a sheet;
- a Contingency Naming Test of executive function designed to assess response inhibition, attentional shift and cognitive flexibility;
- Score, a test of auditory sustained and selective attention in which the child was required to place beads on one of two plates only after a special sound was heard from a cassette tape;
- the People Search, a test of visual sustained and selective attention in which the child was required to cross out compete figures as quickly as possible on a stimulus sheet comprising complete and incomplete stick figures;
- the Coloured Progressive Matrices (CPM) in which matrices of abstract patterns with a missing piece were presented and the child was required to complete the pattern with one from a choice of four pieces. This test assessed non-verbal reasoning and was administered to rule out impairment in global mental functioning.

A detailed description of the tests is presented elsewhere (Kitsao-Wekulo et al., 2012).

To reduce the test battery to a smaller set of ability composites, z-scores for each measure were subjected to principal component factor analysis with Varimax rotation. Based on factor content, skill composites were labeled Executive Function and Verbal Memory. Skill composites of the z-scores comprising each factor were computed based on factor weightings.

### Data collection procedures

All the tests were administered at a school near the child's home. Each child was tested individually in a quiet area within sight of other children, and in familiar surroundings to minimize test anxiety. Observations by the assessors suggested that none of the children was unduly anxious during the test sessions.

### Data analysis

Independent samples *t*-tests, Chi-square tests and univariate analysis were undertaken to determine group differences in nutritional status and outcomes. Pearson product-moment correlation coefficients were used to examine the relationship between the background variables and cognitive outcomes, language skills, motor abilities, and nutritional status. AMOS version 20 (SPSS) was used to test the fit of the overall model and to examine the relationships among the variables. SEM was used to examine the relationships between background characteristics, child nutritional status and child outcomes. We developed and tested a path analysis model (Figure 1) based on logic and theory about how background variables co-vary with nutritional status, and how they influence child outcomes directly and indirectly. In the full model which included all the explanatory variables, this format allowed us to test the mechanisms through which each of the background variables influenced various child outcomes directly and indirectly though a mediated path. An independent disturbance term that represented unexplained variance was estimated for each endogenous variable.

In fitting the Structural Equation Models, missing information was taken into account using the Maximum Likelihood (ML) Estimates. The ML technique assumes data are missing at random for continuous, binary, and categorical variables. All direct and indirect paths were tested and each of the four child outcomes was analyzed in isolation. Specific procedures for model development were to remove non-significant paths (*p* = 0.05) and use modification indices as suggested by the AMOS SEM program (Arbuckle, 1988) to add paths or correlations that would improve model fit. Chi-square analysis was conducted in initial examination of the goodness of fit to insure non-significance. However, because this method is sensitive to sample size, other indices of goodness of fit included the Tucker Lewis Index (TLI), Comparative Fit Index (CFI), and Root Mean Square Error of Approximation (RMSEA) (Bentler and Chou, 1987; Browne and Cudeck, 1993). Acceptable fit was defined as TLI and CFI >0.90 and RMSEA <0.08 and an excellent fit as TLI and CFI >0.95 and RMSEA <0.05.

## Results

### Descriptive statistics

The study involved 308 boys and girls. The prevalence of linear growth retardation in this study population was high. Approximately 24% (*N* = 74) of all the children were stunted. Table 1 portrays a summary of the sample characteristics. The proportion of stunted children residing in rural areas was significantly higher than that of their counterparts in peri-urban areas, χ^2^ (1, *N* = 308) = 4.12, *p* = 0.04. A higher proportion of girls than boys was stunted but these differences were not significant, χ^2^ (1, *N* = 308) = 1.48, *p* = 0.22.

**Table 1:** **Description of sample characteristics, *N* = 308**.

**Variable**	**Stunted**		**Not stunted**	
	***N***	**%**	***N***	**%**
**GENDER**				
Boys	31	20.9	117	79.1
Girls	43	26.9	117	73.1
**AREA OF RESIDENCE**				
Rural	65	26.5	180	73.5
Peri-urban	9	14.3	54	85.7
**AGE (YEARS)**				
≤8.0	11	15.3	61	84.7
8.5–9.0	19	17.6	89	82.4
≥9.5	44	34.4	84	65.6
**SCHOOL EXPOSURE**				
0 years	22	62.9	13	37.1
1–2 years	21	20.8	80	79.2
>2years	31	18	141	82
**HOUSEHOLD WEALTH**				
Level 1	39	31.7	84	68.3
Level 2	21	22.3	73	77.7
Level 3	14	15.4	77	84.6

More than one-third of the oldest children (aged 9.5 years or more) compared to 15.3% in the youngest group (aged 8 years or less) and 17.6% among those aged between 8.5 and 9 years were stunted. These differences were significant, χ^2^ (2, *N* = 308) = 12.98, *p* = 0.002. Among children who did not attend school, a very high proportion was stunted compared to their counterparts who had attended school for at least 1 year and those with more than 2 years of school exposure. These differences were highly significant, χ^2^ (2, *N* = 308) = 32.89, *p* < 0.001. In terms of household wealth, the highest proportion of stunted children was found among those in the sample who were least wealthy (Level 1). The differences in prevalence of stunting among the three groups were significant, χ^2^ (2, *N* = 308) = 7.85, *p* = 0.02.

### Correlations

Variable intercorrelations are presented in Table 2. As can be seen from the table, more schooling and higher age were the most frequently correlated with household wealth, stunting, and child outcomes. These correlations provide some initial evidence that school attendance and age have moderate to strong associations with nutritional status, which in turn is associated with children's language functioning and motor skills.

**Table 2:** **Correlations among variables in the models**.

	**1**	**2**	**3**	**4**	**5**	**6**	**7**	**8**	**9**
Area of residence	1								
Gender	−0.012	1							
Age	−0.025	0.019	1						
Years in school	0.313^**^	−0.084	0.041	1					
HAZ	0.130^*^	−0.006	−0.300^**^	0.272^**^	1				
Household wealth	0.135^*^	−0.067	−0.240^**^	0.391^**^	0.146^*^	1			
Language scores	0.045	−0.166^**^	0.318^**^	0.427^**^	0.127^*^	0.048	1		
Motor scores	0.060	0.074	0.402^**^	0.318^**^	0.106	0.017	0.499^**^	1	
Verbal memory	−0.010	0.134^*^	0.182^**^	0.125^*^	0.043	−0.009	0.259^**^	0.311^**^	1
Executive function	0.213^**^	−0.082	0.28^**^	0.519^**^	0.240^**^	0.107	0.554^**^	0.614^**^	0.397^**^

* p < 0.05;

** p < 0.01.

### Differences in outcomes

Children who were stunted performed more poorly than their counterparts who were not stunted on all the outcomes tested (Table 3). These differences were significant for the tests of language, *t*_(306equal variances_ = −2.627, *p* = 0.009, and executive function, *t*_(100unequal variances)_ = −2.490, *p* = 0.014. (Levene's test indicated unequal variances (*F* = 5.572, *p* = 0.019), so degrees of freedom were adjusted from 306 to 100 for executive function). Medium effect sizes were seen for language and executive function tests.

**Table 3:** **Differences in outcomes**.

	**Stunted (*N* = 74)**		**Not stunted (*N* = 234)**		**Cohen's *d***
	**Mean**	***SD***	**Mean**	***SD***	
Language skills	−0.26	1.09	0.08	0.95	0.333
Motor abilities	−0.06	0.72	0.03	0.57	0.140
Verbal memory	−0.03	0.89	0.01	1.03	0.042
Executive function	−0.26	1.04	0.08	0.83	0.364

### Model modification

**Figure 2:**
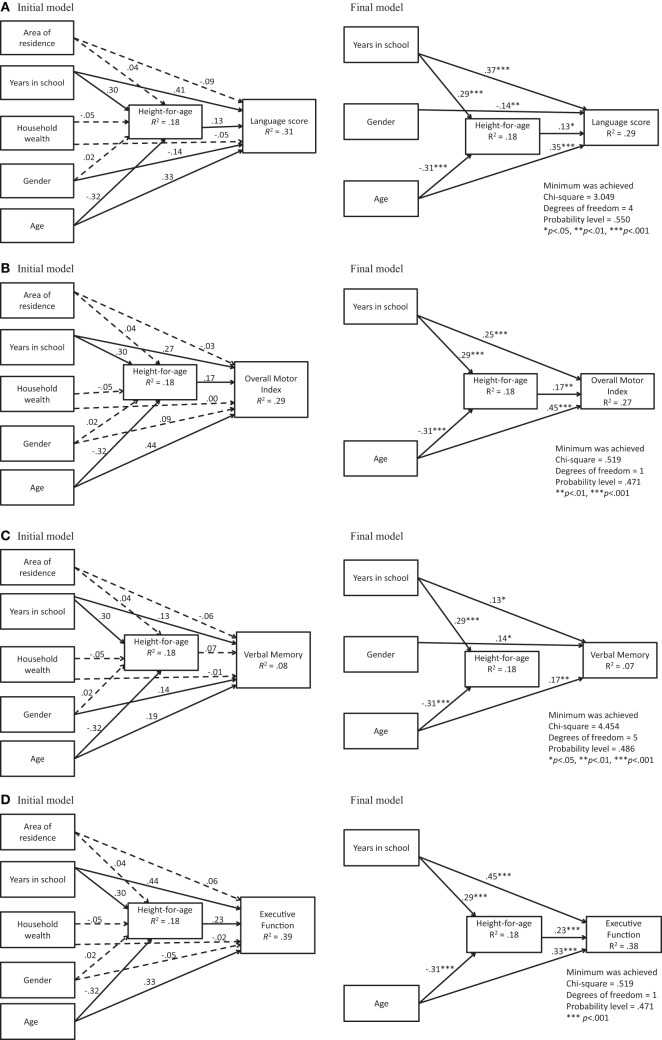
**(A)** Initial and final models for language score. **(B)** Initial and final models for motor skills. **(C)** Initial and final models for verbal memory score. **(D)** Initial and final models for executive function score.

For each outcome, the initial model did not have a good fit. The steps in developing the individual path models involved making several revisions by deleting non-significant paths and covariances (Table 4). (Non-significant paths in initial models are indicated with dashed lines). Modification indices did not suggest the need for additional paths or correlations. The final models for the four child outcomes provided a good fit to the data. In order to simplify the output, only significant standardized path coefficients are shown in the final models (Figures 2A–D).

**Table 4:** **Maximum likelihood estimates of covariances for initial model**.

**Covariance**	**Covariance estimate**	**Standard error**	**Correlation estimate**	***p*-value**
Years in school ↔ Area of residence	0.212	0.041	0.313	<0.001
Age ↔ Household wealth	−1.049	0.257	−0.240	<0.001
Area of residence ↔ Household wealth	0.214	0.091	0.135	0.019
Age ↔ Gender	0.011	0.032	0.019	0.738
Years in school ↔ Age	0.077	0.107	0.041	0.472
Household wealth ↔ Gender	−0.132	0.112	−0.067	0.238
Years in school ↔ Gender	−0.071	0.048	−0.084	0.141
Area of residence ↔ Gender	−0.002	0.012	−0.012	0.837
Age ↔ Area of residence	−0.011	0.026	−0.025	0.657
Years In school ↔ Household wealth	2.584	0.405	0.391	<0.001

#### Language skills

The model for language skills (Figure 2A) fitted well, TLI >0.99, CFI >0.99, RMSEA <0.05. School attendance and age were related directly and indirectly (through nutritional status) to language skills. While more years of being in school were associated with both better nutritional status and higher language scores, associations of nutritional status and outcomes with gender and age were less consistently observed. Younger children had better nutritional status while older children had better language outcomes. Boys had higher language scores than girls. The indirect path from gender through nutritional status was not significant. Direct paths from height-for-age Z-scores to outcome indicated associations of better nutritional status with higher scores on the language test. These results suggest that the influences of school and age (but not gender) on language scores were partially mediated through nutritional status.

#### Motor abilities

The model for motor abilities had an excellent fit, χ^2^ (1, *N* = 308) = 0.519, *p* = 0.47; TLI >0.99, CFI >0.99, RMSEA <0.05 (Figure 2B). Paths linking longer attendance at school and higher age with outcome suggest that these two variables were directly and indirectly associated with motor abilities. Direct paths from height-for-age Z-scores to outcome indicated associations of better nutritional status with higher scores on the motor test.

#### Verbal memory

The model for verbal memory had a good fit, χ^2^ (5, *N* = 308) = 4.45, *p* = 0.49; TLI >0.99, CFI >0.99, RMSEA <0.05, but it explained very little of the variance observed (Figure 2C). School attendance, gender, and age had a direct effect on verbal memory. As with the other models, the association between gender and nutritional status remained non-significant. Gender had a small effect on outcome and this effect favored girls. The path coefficient from nutritional status to verbal memory was not significant.

#### Executive function

The model for executive function also had a good fit, χ^2^ (1, *N* = 308) = 0.519, *p* = 0.47; TLI >0.99, CFI >0.99, RMSEA <0.05 (Figure 2D). School attendance and age showed strong links with executive function indicating associations of more schooling and higher age with higher scores on executive function tests. Moreover, the direct and indirect effects were significant and the path coefficient from nutritional status to executive function was higher than for all other outcomes.

## Discussion

Although the direct effects of poor nutritional status on child neurocognitive functioning have been well-documented in the literature, very little is known about the complexities of that relationship in a multiple risk environment. Through the use of SEM, this study has attempted to elucidate some of the pathways through which nutritional status and other contextual characteristics may influence outcome in school-age children.

The risk factors for poor nutritional status in this population included older age, rural place of residence, low household wealth levels and not attending school. That younger children had a better nutritional status than their older counterparts was not unexpected; similar findings have been reported in earlier studies among infant (Powell and Grantham-McGregor, 1985) and school-age populations (Senbanjo et al., 2011). We also found that the prevalence of stunting was higher in rural than peri-urban areas. As rural areas tend to have high concentrations of people with low education and income levels, children are more likely to suffer the effects of these deprivations, though poorer nutritional status. Fotso (2006), in an effort to compare the magnitude of inequities in child malnutrition in urban and rural areas of selected countries in sub-Saharan Africa, reported similar findings. Moreover, in the current study, children from the least wealthy households faced the greatest risk of being stunted, compared to their counterparts in the most wealthy households, corroborating earlier findings in similar resource-restricted settings (Fotso, 2006). Our finding that levels of stunting were higher among children not attending school could be explained as follows. Children from poor families are more likely to end up with poor nutritional status (Abubakar et al., 2008), and consequently, less likely to attend school (Ivanovic et al., 2012).

In turn, poor nutritional status predicted poorer outcomes on all the tests. These findings are consistent with reports from studies among infants and school-age children living in similar and different contexts (Sigman et al., 1989; Abubakar et al., 2008; Kar et al., 2008; Bangirana et al., 2009). Poor nutritional status results in a wide range of cognitive deficits linked to structural abnormalities of different parts of the brain (Kar et al., 2008). Because stunting occurs in early childhood, these results provide evidence that the effects of poor nutritional status may be long-lasting, especially if appropriate interventions are not put in place.

The data show evidence for associations between background variables and nutritional status, and between nutritional status and multiple cognitive skills. As expected, the paths linking the variables to nutritional status and children's performance differed in magnitude for each outcome. The novelty, level of familiarity with and requirements of the various tasks could perhaps explain the differences observed. Mediated influences of nutritional status, as well as the direct effects of background variables were stronger for tests with a higher degree of novelty, which were less familiar and which had more complicated task requirements. For instance, the requirement to keep a shopping list in memory is a familiar common activity for school-age children. This may be a plausible explanation for the lack of sensitivity to nutritional status influences and weak direct effects observed on verbal memory.

Noteworthy in the current study is the negative relationship between age and nutritional status. Similar patterns have been reported in earlier studies which have recorded a dramatic rise in the prevalence of stunting with age among African children (Stoltzfus et al., 1997; Hautvast et al., 2000; Senbanjo et al., 2011). Stoltzfus et al. (1997) as well as Glewwe and Jacoby (1995) have postulated that, parents probably enrol the more healthy children in school at earlier ages. As a result, a pattern of higher prevalence of poor nutritional status among children who are older emerges. The same situation may pertain to the current study context. Strong age effects were seen on motor skills, language abilities and executive function, a finding which may be attributed to the following. Children's vocabularies expand as their semantic development takes effect (Zembar and Blume, 2009) hence older children do better than younger ones on vocabulary tests. A rapid increase of muscle strength and maturation of physical abilities related to balance and coordination also takes place in middle childhood (Zembar and Blume, 2009) resulting in better performance on motor tests among older children. Also, as this is a particularly active stage of maturation of executive function, children make significant cognitive advancements during middle childhood (Brocki and Bohlin, 2004).

Associations of gender with nutritional status and with motor skills and executive function did not reach significance. The literature on gender differences in nutritional status and gender influences on child outcomes illustrates a non-uniform pattern. Studies in sub-Saharan Africa, for example, report higher levels of stunting among boys (Semproli and Gualdi-Russo, 2007; Wamani et al., 2007; Goon et al., 2011), while studies from elsewhere have recorded higher levels for girls (Chowdbury et al., 2008). Although the literature on malnutrition seems to suggest that the differences in the manner in which boys and girls are treated may help one gender overcome early adversity, this did not seem to be the case in the current study. Our study also revealed that boys achieved higher scores on the language test while the reverse was true for verbal memory. Contrasting findings have, however, been reported in other studies where girls are found to consistently outperform boys on both measures (Kramer et al., 1997; Lowe et al., 2003). Perhaps in their day to day interactions, boys had more extensive prior experience with the objects that were represented pictorially on the language test hence they had an advantage over girls in naming the items. On the other hand, superior verbal memory scores for girls may be attributed to earlier maturation of their brains.

Our index for household wealth did not have significant direct or indirect effects on any of the child outcomes. On the contrary, several studies have reported that socioeconomic status is a strong predictor of both nutritional status (Brooks-Gunn and Duncan, 1997; Ndukwu et al., 2013) and outcomes in children (Bradley and Corwyn, 2002; Santos et al., 2008). The lack of an association between household wealth and child outcomes is not without precedence; an earlier study among infants living within the same context (Abubakar et al., 2008) has reported similar findings. We offer a couple of explanations for the non-significant direct effects of household wealth on nutritional status and child outcomes. First, we speculate that this finding may relate to the overwhelming influence of other factors, such as school attendance, among children at this age. This is evidenced by the moderate correlation seen between household wealth and school attendance. Secondly, our study was conducted within a context in which the majority of families live in economically depressed conditions. This may be the reason why, even though the indicators included in our SES measure distinguished one household from another, these differences were not significant in relation to the outcomes under study.

Although other studies have reported that children residing in rural areas have a substantially higher risk of poor nutritional status compared to their urban counterparts (Hautvast et al., 2000; Fox and Heaton, 2012), our study did not show evidence of such associations. The primary reason for this finding was that the current study was conducted within a predominantly rural context. Variations in children's area of residence may therefore have been too subtle to create any real differences in outcomes for children.

In the final trimmed models, school attendance had both direct and indirect (via nutrition) effects and was the most influential environmental predictor of nutritional status and child outcomes. The possibility that the nutrition-related benefits afforded by a school feeding program may explain this finding was negated by the fact that it was only in one school that children were offered food in school. When school attendance was taken into account, associations of nutritional status and cognitive functions with demographic factors like household wealth lost their significance; any bivariate associations washed out with the effects of going to school. This finding provides evidence that school attendance captures family resources more globally and meaningfully (such that there were no independent effects of area of residence and household wealth). Our models are also consistent with earlier studies that have demonstrated that where school attendance is not universal, even a little school exposure is associated with improved test-taking performance. In part, this may be due to increased test-taking awareness, as well as to methods of instruction, curriculum content or the types of questions that teachers ask, accelerating the development of cognitive skills over and above other factors (Holding et al., 2004; Alcock et al., 2008). Going to school thus offers opportunities for learning and practice, and also trains children to follow instructions, hence the strong associations observed with tasks of higher order functioning.

Building up on previous similar work in this area, similarities were seen in the magnitude of the associations between background variables and nutritional status. However, the relationship between SES, stunting and outcome seen among infants (Abubakar et al., 2008) within the same context was not fully replicated in the current study population. This may have been because older children are exposed to more varied environments. Furthermore, as with the infant study, the direct path between household wealth and outcome in our study was not significant. As reiterated earlier on in this discussion, school attendance seemed to exert a greater influence than household wealth on nutritional status, and had strong direct associations with all outcomes (except verbal memory). A plausible explanation for this finding is that by the time children attain the age of going to school (around 6 years in the study context), the individual effects of socioeconomic status diminish as household wealth becomes an important determinant of whether or not a child goes to school (Mishra et al., 2007). Parents who are doing relatively well economically are able both provide more nutritious meals for their children as well as to retain their children in school. On the other hand, poor nutritional status may reflect limited economic resources. School attendance patterns of children from less wealthy households may be characterized by prolonged absenteeism or dropouts as their parents are unable to initiate and maintain their children's schooling (Mendez and Adair, 1999). Such children are thus likely to benefit less from the effects of school exposure. In light of these associations, school attendance could therefore be considered a proxy for household wealth, which in turn is strongly related to the nutritional status of the child. That there is a complex interactive relationship among the three factors is supported by the suggestion by Mukudi (2003) that the association between school attendance and nutritional status is a function of socioeconomic status. These associations could be more extensively explored through a longitudinal study.

Some of the major difficulties that emerge when comparing the effects of background variables on child development in different populations arise from the differences in environments to which they are exposed and in the outcomes tested. As noted by Goon et al. (2011), historical data such as birth weight, birth order, duration of breastfeeding and birth interval would likely provide a picture of previous states of malnutrition and provide further understanding of its aetiology within the current study population.

The estimated models demonstrated the continued importance of nutritional status as a powerful predictor of outcomes even as children grow older. Significant direct effects of the background variables on child outcomes suggest that the estimated models do not fully explain pathways through which they might influence child outcomes. The unexplained variance may be found in the home environment, an area which remains poorly investigated among rural African populations. Interventions to ameliorate the negative effects of poor nutritional status earlier on may mitigate the need for costly interventions later on, especially for those growing up in the contexts of poverty and poor nutrition.

### Conflict of interest statement

The authors declare that the research was conducted in the absence of any commercial or financial relationships that could be construed as a potential conflict of interest.
